# Interpreting the different emissive properties of cyclic tri­imidazole-based Cu^I^ and Ag^I^ coordination polymers: a QTAIM and IQA study

**DOI:** 10.1107/S2052520621009707

**Published:** 2021-11-05

**Authors:** Alessandra Forni, Elena Cariati, Lucia Carlucci, Elena Lucenti, Daniele Marinotto, Stefano Pieraccini, Maurizio Sironi

**Affiliations:** a CNR-SCITEC, Institute of Chemical Sciences and Technologies "Guilio Natta" and INSTM RU, via Golgi 19, 20133 Milano, Italy; b Department of Chemistry, Università degli Studi di Milano and INSTM RU, via Golgi 19, 20133 Milano, Italy

**Keywords:** Ag^I^ and Cu^I^ coordination polymers, room-temperature phospho­rescence, metal–ligand interaction, QTAIM, IQA, source function, metal–halogen bond, quantum crystallography

## Abstract

A QTAIM and IQA investigation on model compounds of two isostructural Ag^I^ and Cu^I^ coordination polymers (CPs) based on cyclic tri­imidazole (*L*), *i.e.* the [*M*I*L*]_*n*_ 1D double-stranded stair chain and the [*M*Cl*L*]_*n*_ 3D network (*M* = Cu, Ag), has allowed light to be shed on the different emissive behaviour associated with the two metal ions. According to a previously reported investigation [Malpicci *et al.* (2021). *Inorg. Chem. Front.* **8**, 1312–1323 ▸], Ag^I^ CPs showed both fluorescence and multiple ligand-centred room-temperature phospho­rescences, whereas Cu^I^ CPs displayed non-thermally equilibrated halogen and metal-to-ligand charge transfer and two ligand-centred phospho­rescences, the latter observed only by their selective activation. Analysis of both local and integral QTAIM descriptors, including delocalization indices and source function, of the Ag—N and Cu—N bonds reveals a higher covalent and local character for the latter, explaining the greater metal–ligand electronic communication observed for the Cu compounds. Moreover, IQA investigation shows that the Cu—N bond is characterized by higher interaction energy, due to both higher electrostatic and exchange-correlation contributions. Analysis on the *M*—*X* (*M* = Ag, Cu; *X* = I, Cl) bonds, also present in these structures, highlights a much higher covalent and local character with respect to the *M*—N bonds.

## Abstract 2

The different nature of the emissive states of Ag^I^ and Cu^I^ one- and three-dimensional coordination polymers is here explained by a higher covalent character of the Cu—N bond with respect to the Ag—N one.

## 1. Introduction

The development of new materials with selected properties and improved performance strongly relies on the understanding of the nature of chemical interactions governing their structure. A formidable tool to rationalize chemical bonding is represented by the electron density distribution, an observable which can be derived from both quantum-mechanical calculations and experimental methods, the latter generally based on X-ray diffraction techniques. Analysis of electron density is mainly performed within the Bader’s Quantum Theory of Atoms In Molecules (QTAIM) approach (Bader, 1990 ▸), which offers a number of local and integral descriptors to characterize chemical bonding. A recent review (Tolborg & Iversen, 2019 ▸) extensively illustrated how electron density studies can provide deep understanding of the relationships between chemical bonding and properties for several classes of materials, including thermoelectric materials, high-pressure electrides and materials for nonlinear optics.

In this paper, we propose to extend this kind of investigation to the field of photoluminescent materials. Some of us have recently reported on the emissive properties of isostructural Cu^I^ (Lucenti *et al.*, 2019 ▸) and Ag^I^ (Malpicci *et al.*, 2021 ▸) coordination polymers (CPs) based on the cyclic tri­imidazole ligand (*L*, see Scheme 1), *i.e.* the [*M*I*L*]_*n*_ 1D double-stranded stair chain and the [*M*Cl*L*]_*n*_ 3D network (*M* = Cu, Ag). The cyclic tri­imidazole, triimidazo[1,2-*a*:1′,2′-*c*:1″,2″-*e*][1,3,5]-triazine (see Scheme 1), was previously shown (Lucenti *et al.*, 2017 ▸) to display aggregation-induced emission behaviour (Luo *et al.*, 2001 ▸; Mei *et al.*, 2015 ▸) revealing, in particular, ultralong phospho­rescence (1s) under ambient conditions associated with the presence of strong π–π stacking interactions in its crystal structure. 
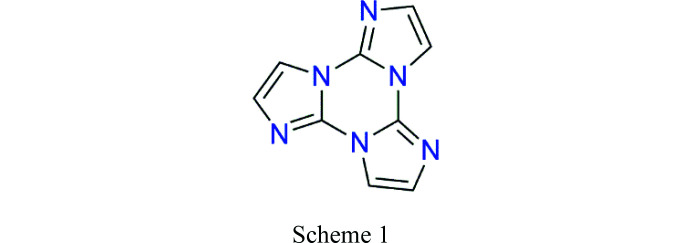


As reported by Malpicci *et al.* (2021 ▸), [*M*I*L*]_*n*_ and [*M*Cl*L*]_*n*_ CPs were found to exhibit quite different properties according to the metal atom, though the associated overall quantum yields, measured for [*M*I*L*]_*n*_ compounds (19 and 18% for the Ag and the Cu derivatives, respectively), were found to be comparable. In particular, Ag^I^ CPs showed at room-temperature (RT) both fluorescence (at 400 and 448 nm for [AgI*L*]_*n*_ and [AgCl*L*]_*n*_ CPs, respectively) and multiple vibrationally resolved phospho­rescences, the latter simultaneously activated and ascribed, based on X-ray structural analysis and DFT/TDDFT calculations on discrete models of the CPs, to π–π stacking interactions of the ligand (T_H_-S_0_, main peaks at 530 and 526 nm for [AgI*L*]_*n*_ and [AgCl*L*]_*n*_ CPs, respectively), ligand-centred emissive states (T_M_-S_0_, main peaks at 411 and 520 nm for the two compounds, respectively) and, only for [AgI*L*]_*n*_, intermolecular electronic coupling by an extrinsic heavy-atom effect (T_I_-S_0_, at about 480 nm). Emission spectra of Cu^I^ CPs at RT, on the other hand, were dominated by a XMLCT (halogen and metal-to-ligand charge transfer) unresolved phospho­rescence (T_M_-S_0_, at 568 and 560 nm for [CuI*L*]_*n*_ and [CuCl*L*]_*n*_ CPs, respectively). Additional resolved long-lived emissions, non-thermally equilibrated with the XMLCT one, were observed only by their selective activation (T_H_-S_0_, main peaks at 536 and 515 nm for [CuI*L*]_*n*_ and [CuCl*L*]_*n*_ CPs, respectively; T_I_-S_0_ for [CuI*L*]_*n*_, main peak at 460 nm). No fluorescent emission was detected for Cu compounds. Moreover, quite different lifetimes were measured for the ligand-centred T_H_-S_0_ phospho­rescence, much longer for the Ag^I^ (τ_av_ ∼ 40 and 48 ms for [AgI*L*]_*n*_ and [AgCl*L*]_*n*_ CPs, respectively) than for the Cu^I^ compounds (τ_av_ ∼ 0.3 and 4 ms for [CuI*L*]_*n*_ and [CuCl*L*]_*n*_ CPs, respectively).

**Figure 1:**
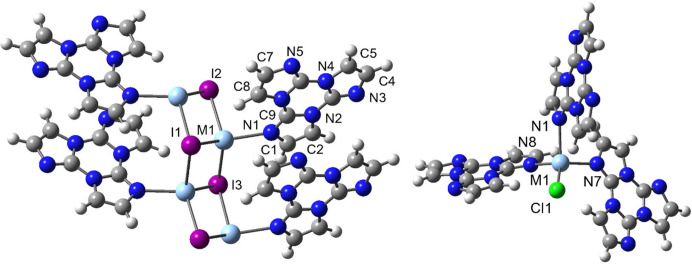
[*M*I*L*]_4_ (left) and *M*Cl*L*_3_ (right) (*M* = Ag, Cu) model compounds. Atoms of the ligand including N1 in *M*Cl*L*_3_ are labelled as in [*M*I*L*]_4_.

All this experimental evidence pointed to a different nature of the Ag—N and Cu—N bonds connecting the metal with the ligand, suggesting a greater electronic communication for the latter bond which results in a more facile singlet-to-triplet intersystem crossing despite the lower atomic weight of the metal. While the small dimensions of the [*M*I*L*]_*n*_ and [MCl*L*]_*n*_ crystals precluded the collection of the high-resolution data required for experimental charge density studies, preliminary results of QTAIM topological analysis on the wavefunction of [*M*I*L*]_4_ model compounds (see Fig. 1 ▸, left) were reported by Malpicci *et al.* (2021 ▸). According to this analysis, greater covalent character was obtained for the Cu—N bond with respect to the Ag—N one, as indicated, in particular, by a higher value of delocalization index (DI), *i.e.* the average number of electrons shared between *M* and N (Daudel *et al.*, 1974 ▸; Bader & Stephens, 1975 ▸).

Herein, after a brief reminder of the main structural features of the investigated systems, we report a more comprehensive QTAIM study of the *M*—N bond in the investigated compounds by (i) analyzing an additional topological descriptor such as the source function (Bader & Gatti, 1998 ▸) and the energetic contributions to the *M*—N bonds based on the Interacting Quantum Atom Approach (IQA) (Blanco *et al.*, 2005 ▸), and (ii) extending the study to *M*Cl*L*_3_ (see Fig. 1 ▸, right), the model compound of [*M*Cl*L*]_*n*_, to further validate our conclusions on a different compound. In addition, QTAIM and IQA analyses on other relevant bonds and intramolecular interactions present in these structures are included.

## 2. Experimental

As reported by Lucenti *et al.* (2019 ▸) and by Malpicci *et al.* (2021 ▸), [*M*I*L*]_*n*_ and [*M*Cl*L*]_*n*_ CPs (*M* = Ag and Cu) crystallize in space groups *P*2_1_/*c* and 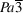 , respectively. Their asymmetric units contain one metal atom, one halogen atom and one tri­imidazole ligand (for [*M*I*L*]_*n*_) or one third of it (for [*M*Cl*L*]_*n*_). In [*M*I*L*]_*n*_, metal atoms are coordinated to three μ_3_-I ions [*M*—I distances equal to 2.8084 (5), 2.8682 (5), 2.9221 (5) Å and 2.6107 (5), 2.7231 (4), 2.7716 (5) Å for *M* = Ag and Cu, respectively] and one monodentate tri­imidazole ligand [*M*—N distances equal to 2.319 (3) and 2.0284 (19) Å for *M* = Ag and Cu, respectively] in an *M*I_3_N distorted tetrahedral environment. The ligands on both sides of the chains, placed at interplanar distance of 3.1726 (19) (*M* = Ag) and 3.1730 (6) Å (*M* = Cu), are involved in strong π–π stacking interactions. Adjacent chains, connected through weak C—H⋯N interactions, are separated by I⋯C contacts [3.664 (4) and 3.721 (3) Å for *M* = Ag and Cu, respectively] comparable with the sum of the relevant van der Waals radii. According to Malpicci *et al.* (2021 ▸), the interligand π–π interactions and the I⋯C contacts were deemed responsible, respectively, for the T_H_-S_0_ and T_I_-S_0_ radiative deactivations observed for [*M*I*L*]_*n*_ CPs.

In [*M*Cl*L*]_*n*_, the metal atoms are in an *M*ClN_3_ distorted tetrahedral environment, coordinating three tridentate tri­imidazole ligands and one terminal chloride anion [*M*—N = 2.354 (1) and 2.130 (1) Å; *M*—Cl = 2.4562 (5) and 2.2567 (6) Å, for *M* = Ag and Cu, respectively]. The resulting structure consists of two interpenetrated three-connected 3D networks of chiral **srs** topology (O’Keeffe *et al.*, 2008 ▸), giving rise to a centrosymmetric framework where tri­imidazole ligands belonging to different srs networks are placed at interplanar distances of 3.3281 (4) (*M* = Ag) or 3.3135 (3) Å (*M* = Cu). Also in this case, the associated π–π interactions were deemed responsible for the T_H_-S_0_ radiative deactivation observed for [*M*Cl*L*]_*n*_ CPs.

Starting from the crystal structures of [*M*I*L*]_*n*_ and [*M*Cl*L*]_*n*_ CPs, the geometries of [*M*I*L*]_4_ and *M*Cl*L*_3_ model compounds (see Fig. 1 ▸) were optimized at the ωB97X/def2-TZVP and ωB97X/6-311++G(d,p) levels of theory for the Ag and the Cu compounds, respectively [see Malpicci *et al.* (2021 ▸)], using *Gaussian16* (Frisch *et al.*, 2016 ▸). The ωB97X functional was chosen in view of its optimal performance in treating the geometrical and electronic features of tri­imidazole derivatives (Lucenti *et al.*, 2017 ▸), including π–π interactions that play an important role in the photophysics of the present structures. For [*M*I*L*]_4_, only bond distances were optimized, while valence angles and torsion angles were frozen to preserve the correct coordination geometry around the metal ions. As expected, the symmetry of the extended polymeric chain was lost in the tetrameric model owing to major boundary effects, resulting in slightly different interatomic distances for equivalent (in the real crystal structure) bonds, in particular for the Cu compounds. Therefore, when discussing geometrical, QTAIM and IQA properties, we will refer to a specific subunit, as labelled in Fig. 1 ▸, keeping in mind that very similar results are obtained for equivalent bonds. For *M*Cl*L*_3_, on the other hand, geometry optimizations did not require the use of constraints, and symmetric structures with virtually equal interatomic distances have been obtained for equivalent bonds. The QTAIM and IQA analyses were performed by *AIMAll* (Keith, 2012 ▸) using, differently from Malpicci *et al.* (2021 ▸), the M06-2X wavefunction, accounting for the fact that this functional, unlike ωB97X, is supported in this software for the calculation of atomic DFT-based exchange-correlation energies in additive IQA atomic energies. To evaluate the effect of the adopted functional on the QTAIM properties where such terms do not contribute, we will report, only for these ones, the results previously obtained with ωB97X. For details about the approximations adopted in the DFT implementation in *AIMAll*, the related documentation should be referred (Keith, 2012 ▸).

## 3. Results and discussion

As shown in Table 1 ▸, *M*—N and *M*—*X* bond lengths (*X* = I, Cl), if compared with the X-ray values (see *Experimental* [Sec sec2]) are slightly overestimated by calculations, mainly owing to the lack of crystal packing effects on the computed structures. The discrepancies are greater for the Ag derivatives due to the required pseudopotential-based basis set used to treat these compounds. The *M*—N bonds show relatively low electron densities (ρ_BCP_) and small and positive Laplacian values (∇^2^ρ_BCP_) at the bond critical points (BCPs), suggesting to a first approximation a close-shell nature for these bonds. However, they are characterized by an ‘incipient covalence’ (Espinosa *et al.*, 2002 ▸; Macchi & Sironi, 2003 ▸; Gatti, 2005 ▸) owing to negative *H*_BCP_/ρ_BCP_ ratios, |*V*_BCP_|/*G*_BCP_ values greater than 1 and rather high delocalization indices DI, being *V*_BCP_, *G*_BCP_ and *H*_BCP_ the potential, kinetic and total energy density at the BCP, respectively. Each topological property assumes slightly higher values in [*M*I*L*]_4_ than in *M*Cl*L*_3_ mainly due to the different halogen/ligand ratios in the coordination environments (3:1 versus 1:3, respectively). Altogether, the values of the QTAIM properties for the *M*—N bond are in line with those reported in literature for the few studies on metal–nitro­gen bonds (Cukrowski *et al.*, 2014 ▸; Thomsen *et al.*, 2015 ▸). It should be noted that, compared with results obtained with the M06-2X wavefunction, the *H*_BCP_/ρ_BCP_, |*V*_BCP_|/*G*_BCP_ and DI values previously determined with the ωB97X wavefunction, also reported in Table 1 ▸ (second line) are systematically higher, with even significant differences regarding in particular the delocalization indices. This suggests some caution when comparing topological properties obtained for different bonds with different wavefunctions.

Comparing the same compound bearing Ag or Cu atom, it is evident a markedly greater covalent character for the Cu—N bond with respect to the Ag—N one, systematically reproduced in both compounds. Such a result is particularly reliable when considering integral descriptors such as the DI(*M*,N) values rather than local BCP properties. Further insight into the nature of the Ag—N and Cu—N bonds, and, in particular, on the degree of electronic communication between the two atoms, is provided by the source function (SF) analysis at their respective BCPs (Bader & Gatti, 1998 ▸; Gatti, 2012 ▸), which can reveal how local or non-local this interaction is (Gatti *et al.*, 2003 ▸). Moreover, considering homonuclear bonds, more covalently bonded are the bonded atoms, higher will be their related SF% contribution, owing to greater ability to contribute to the electron density value at their intervening BCP (Gatti *et al.*, 2003 ▸). Looking at the SF% values at the *M*1—N1 BCPs (see Tables 2 ▸ and 3 ▸ for [*M*I*L*]_4_ and *M*Cl*L*_3_ compounds, respectively), it appears that the bonded atoms contribute by only 53.6, 50.0% (Ag derivatives) and 66.0, 55.4% (Cu derivatives) to the total density, denoting rather non-local interactions in particular for the Ag derivatives (compare these values with, for example, those obtained for N1—C1, 83.9%, and C1—C2, 84.1%). The remaining contributions essentially come from the other atoms of the bonded ligand, mainly those closer to N1. Interestingly, the metal atom contributes in rather the same way (about 40%) to the total density at the *M*1—N1 BCP, be it Ag or Cu. Moreover, by summing all contributions coming from the ligand, approximately the same SF% value (about 50%) is obtained for all the four compounds. It is therefore concluded that the higher local character of Cu—N with respect to Ag—N is essentially due to the bonded nitro­gen atom, whose contribution to the total density at the Cu—N BCP is systematically greater (by about 11 and 7% for [*M*I*L*]_4_ and *M*Cl*L*_3_ compounds, respectively) than that at the Ag—N BCP, at the expense of the other atoms of the ligand. On the other hand, by comparing the same *M*—N bond in [*M*I*L*]_4_ and *M*Cl*L*_3_, a greater local character emerges for the former compounds, in agreement with a greater covalency as depicted by the DI(*M*,N) values (see Table 1 ▸).

According to the IQA method (Blanco *et al.*, 2005 ▸), binding energies relative to a given reference state (*e.g.* the isolated neutral atoms or molecules) may be expressed in terms of the changes in the self-energies of the interacting fragments with respect to their values in the reference state, and in terms of the pair-wise interaction energies between fragments. The latter are made up of classical electrostatic components between atoms *A* and *B* (*V*_cl_^*AB*^) and of a stabilizing quantum-mechanical contribution (*V*_XC_^*AB*^). These two contributions, providing the interaction energy for the *A*–*B* pair, *E*_int_^*AB*^, are reported in Tables 4 ▸ and 5 ▸ for selected bonded and non-bonded atoms of [*M*I*L*]_4_ and *M*Cl*L*_3_, respectively. The results indicate, for both compounds, a higher interaction energy for the Cu—N bond, due to both higher electrostatic and exchange-correlation contributions. The higher *V*_XC_^*AB*^ values obtained for the Cu—N bond agree with the higher electronic communication as depicted by the above reported QTAIM values. Interestingly, however, it is observed that the *V*_XC_^*AB*^ contributions with respect to the total interaction energy are essentially the same, independently on both the compound and the *M*—N bond.

The QTAIM results obtained from the present investigation on Cu—N and Ag—N bonds roughly parallel those previously derived for C—Br and C—I, *i.e.* other bonding interactions involving a light atom and fourth or fifth-row heavy atoms of the same group, through an extensive charge density study on isostructural complexes of (*E*)-1,2-bis­(4-pyridyl)­ethyl­ene with 1,4-dihalotetra­fluoro­benzene (Forni, 2009 ▸; Bianchi *et al.*, 2003 ▸). Though the latter bonds, involving non-metal atoms, represent of course much more covalent interactions with respect to the metal–nitro­gen ones [for example, X-ray-based *H*_BCP_/ρ_BCP_ values were −0.91 (2) and −0.63 (2) for C—Br and C—I, respectively, against −0.16 and −0.07 for Cu—N and Ag—N, respectively], the topological descriptors were found to vary in rather the same way going from the fourth to the fifth-row atom, namely a decreasing covalence degree was obtained for increasing atomic weight moving along the same group. As elucidated in previous investigation (Forni, 2009 ▸), this feature should be ascribed to the different electron density distribution around the heavy atom. In fact, based on the topological analysis of the Laplacian of electron density in the valence shell charge concentration region, a much more structured electron density distribution was depicted for the bromine atom with respect to a rather flat one surrounding the iodine atom, allowing to establish more shared-shell (*i.e.* covalent) bonding interactions.

The QTAIM and IQA properties of *M*—*X* (*X* = I, Cl) bonds in [*M*I*L*]_4_ and *M*Cl*L*_3_ are also reported in Tables 1 ▸–5 ▸. It is found that these bonds, while characterized by lower density at their BCP, result to be more covalent with respect to the *M*—N ones, as indicated by all topological descriptors (see Table 1 ▸), with the only exceptions of the weak Cu1—I2 and Cu1—I3 bonds, whose DI values (0.408 and 0.335) are lower than that of Cu1—N1, 0.416. Looking at the SF% values at the *M*—*X* BCPs (see Tables 2 ▸ and 3 ▸), it results that the contributions from the metal atom are slightly lower than (in [*M*I*L*]_4_) or comparable with (in *M*Cl*L*_3_) the values obtained for the *M*—N BCPs. On the other hand, those deriving from the halogen (> 40%) are much greater than the one coming from the nitro­gen atom. As a result, the cumulative SF% contributions from the bonded atoms to the density at the *M*—*X* BCPs are much greater than those obtained for the *M*—N bonds, mirroring the higher covalence character depicted by the *H*_BCP_/ρ_BCP_, |*V*_BCP_|/*G*_BCP_ and DI descriptors. Comparing the different covalence degree of Cu—*X* and Ag—*X* bonds, it is found that all the values of *H*_BCP_/ρ_BCP_ and |*V*_BCP_|/*G*_BCP_ confirm the conclusions drawn for *M*—N bonds, that is, larger values of these descriptors are obtained for Cu—*X* bonds with respect to those of the corresponding Ag—*X* ones, denoting more covalent interactions for the lighter metal atom. However, it is noted that DI(*M*,*X*) follow the same trend only for the stronger *M*1—I1 bond, while the weaker *M*1—I2, *M*1—I3 and *M*1—Cl1 interactions are characterized by lower delocalization indices for the copper derivatives.

In Tables 4 ▸ and 5 ▸, the results of IQA calculations for *M*⋯*M*, *X*⋯N and N⋯N non-bonding interactions are also reported. These contacts are repulsive interactions and, in agreement with the absence of a bond path connecting the two atoms, the associated delocalization indices are <0.1. The computed V_XC_^AB^, however, are not negligible and in some cases even larger than the values obtained for non-bonding interactions for which a bond path was found (for example F⋯F interactions, see Forni *et al.*, 2019 ▸). In particular it is worth noting the high (−7.8 kcal mol^−1^) exchange-correlation contribution for the Ag⋯Ag contact, having delocalization index DI = 0.076, where the absence of an associated BCP was rather unexpected owing to the intermetallic distance, 3.1591 (5) Å in the [AgI*L*]_*n*_ X-ray structure and 3.228 Å in the computed model, significantly shorter than twice the van der Waals radius of silver (3.44 Å) [see Malpicci *et al.* (2021 ▸) for a detailed discussion on this point].

## 4. Conclusion

QTAIM and IQA analyses on the electron density of model compounds of two isostructural Ag and Cu CPs, the [*M*I*L*]_*n*_ 1D double-stranded stair chain and the [*M*Cl*L*]_*n*_ 3D network, are here reported, allowing us to gain useful insight into the nature of Ag—N and Cu—N bonds. Though both bonds can be described as rather non-local interactions, as shown by analysis of the source function, a greater covalent character is shown for Cu—N, based on several QTAIM descriptors including in particular the delocalization indices. Moreover, the Cu—N bond turns out to be a more localized interaction with respect to the Ag—N one, explained by a greater ability of the nitro­gen atom to contribute to the electron density value at their corresponding BCPs. These results can explain the quite different emissive behaviour observed for isostructural Ag and Cu coordination polymers. In fact, photoluminescence spectra of Cu compounds are dominated by an intense XMLCT phosphorescence band due to the easier metal–ligand electronic communication as indicated by the greater covalent character of the Cu—N bond. On the other hand, Ag compounds display fluorescence and multiple ligand-centred phosphorescences, denoting a minor involvement of the metal both in the inter-system crossing process, in spite of its higher atomic weight, and in the nature of the emissive states. While the metal–metal bond has been the subject of several charge density studies, by both experimental and theoretical methods, the metal–ligand bond has received much less attention, with the only exception of the metal—CO bond. We deem that further work in this direction could represent a great challenge for quantum crystallography (Macchi, 2017 ▸).

## Figures and Tables

**Table 1:** Selected bond distances and BCP properties (electron density, ρ_BCP_; Laplacian of electron density, ∇^2^ρ_BCP_; potential, kinetic and total energy density, *V*_BCP_, *G*_BCP_ and *H*_BCP_, respectively, and delocalization index, DI) for [*M*I*L*]_4_ and *M*Cl*L*_3_ (*M* = Ag, Cu) model compounds Wavefunction optimized at ωB97X/def2-TZVP and ωB97X/6-311++G(d,p) levels of theory for the Ag and the Cu compounds, respectively; QTAIM properties computed at M06-2X/def2-TZVP and M06-2X/6-311++G(d,p) (first line) and at ωB97X/def2-TZVP and ωB97X/6-311++G(d,p) levels of theory (second line, in italic), when available, for the Ag and the Cu compounds, respectively.

	*r* (Å)	ρ_BCP_ (e Å^−3^)	∇^2^ρ_BCP_ (e Å^−3^)	*H*_BCP_/ρ_BCP_	|*V*_BCP_|/*G*_BCP_	DI(*M*,N)
[*M*I*L*]_4_						
Ag1—N1	2.382	0.321	5.0	−0.07	1.06	0.329
		*0.328*	*4.8*	*−0.08*	*1.08*	*0.353*
Cu1—N1	2.048	0.494	8.7	−0.16	1.12	0.416
		*0.506*	*8.4*	*−0.17*	*1.13*	*0.457*
Ag1—I1	2.915	0.241	2.2	−0.13	1.17	0.449
		*0.248*	*2.0*	*−0.14*	*1.20*	*0.487*
Ag1—I2	2.937	0.230	2.1	−0.12	1.16	0.431
		*0.237*	*2.0*	*−0.13*	*1.18*	*0.466*
Ag1—I3	2.945	0.226	2.1	−0.12	1.15	0.424
		*0.234*	*1.9*	*−0.13*	*1.18*	*0.460*
Cu1—I1	2.635	0.324	2.4	−0.30	1.36	0.492
		*0.341*	*2.1*	*−0.31*	*1.42*	*0.556*
Cu1—I2	2.757	0.260	1.9	−0.26	1.33	0.408
		*0.274*	*1.7*	*−0.27*	*1.38*	*0.461*
Cu1—I3	2.902	0.199	1.6	−0.20	1.27	0.335
		*0.209*	*1.4*	*−0.21*	*1.30*	*0.375*
*M*Cl*L*_3_						
Ag1—N1	2.426	0.294	4.5	−0.07	1.06	0.315
Cu1—N1	2.167	0.378	6.1	−0.17	1.13	0.345
Ag1—Cl1	2.448	0.413	5.4	−0.17	1.15	0.611
Cu1—Cl1	2.274	0.445	5.7	−0.24	1.21	0.570

**Table 2:** Source function contributions (in %) from relevant atoms to the total density at the *M*—N and *M*—I BCPs for [*M*I*L*]_4_ (*M* = Ag, Cu) model compounds Wavefunction optimized at ωB97X/def2-TZVP and ωB97X/6-311++G(d,p) levels of theory for the Ag and the Cu compounds, respectively; source function computed at M06-2X/def2-TZVP and M06-2X/6-311++G(d,p) levels of theory, for the Ag and the Cu compounds, respectively.Only contributions from atoms of the subunit labelled in Fig. 1 ▸, contributing by more than 1% to the *M*—N BCP of [AgI*L*]_4_ and/or [CuI*L*]_4_, are reported.

	*M*1	N1	I1	I2	I3	N2	N3	N5	C1	H1	C2	H2	C7	H7	C9
Ag1—N1	39.6	14.0	2.3	2.7	2.1	3.9	1.5	3.3	4.9	3.3	4.7	2.2	2.0	1.1	5.6
Cu1—N1	40.8	25.2	2.3	2.5	1.3	2.7	1.0	2.1	3.7	2.4	3.2	1.5	1.3	0.8	4.2
Ag1—I1	33.3	−5.4	42.9	4.6	3.6	1.6	1.2	3.1	0.5	0.7	2.0	1.2	1.5	0.8	0.6
Ag1—I2	32.4	−7.1	4.3	44.9	2.5	1.8	1.3	3.1	0.03	1.0	2.4	1.6	1.5	1.0	0.5
Ag1—I3	32.4	−6.1	3.9	3.2	43.6	1.9	0.8	2.4	−1.0	−1.0	2.8	1.5	1.1	0.7	1.5
Cu1—I1	33.5	−3.6	44.9	4.3	2.7	1.5	1.0	2.5	0.6	0.8	1.7	1.1	1.3	0.7	0.7
Cu1—I2	29.1	−5.7	5.3	45.8	2.0	2.0	1.2	3.0	0.3	1.3	2.5	1.6	1.5	1.0	0.9
Cu1—I3	24.3	−6.2	6.2	4.5	41.1	2.5	1.0	3.0	−1.1	−0.6	3.7	2.0	1.4	1.0	2.3

**Table 3:** Source function contributions (in %) from relevant atoms to the total density at the *M*—N and *M*—Cl BCPs for *M*Cl*L*_3_ (*M* = Ag, Cu) model compounds Wavefunction optimized at ωB97X/def2-TZVP and ωB97X/6-311++G(d,p) levels of theory for the Ag and the Cu compounds, respectively; source function computed at M06-2X/def2-TZVP and M06-2X/6-311++G(d,p) levels of theory, for the Ag and the Cu compounds, respectively.Only contributions from atoms of the subunit labelled in Fig. 1 ▸, contributing by more than 1% to the *M*—N BCP of *M*Cl*L*_3_ compounds, are reported.

	*M*1	N1	Cl1	N7	N8	N2	N3	N5	C1	H1	C2	H2	C7	H7	C9
Ag1—N1	38.1	11.9	4.5	−5.7	−5.4	4.3	1.6	3.6	5.8	3.4	5.2	2.4	2.2	1.3	6.0
Cu1—N1	36.2	19.2	4.6	−4.0	−4.3	3.4	1.2	2.7	4.8	2.8	4.1	1.9	1.6	1.0	5.1
Ag1—Cl1	41.4	−3.3	49.1	−3.3	−3.3	1.0	0.8	1.9	0.5	0.4	1.2	0.8	0.9	0.6	0.3
Cu1—Cl1	38.1	−3.2	49.3	−3.2	−3.2	1.1	0.7	1.8	0.6	0.5	1.3	0.8	1.0	0.6	0.5

**Table 4:** Selected IQA total (*E*_int_^*AB*^), electrostatic (*V*_cl_^*AB*^) and exchange-correlation (*V*_xc_^*AB*^) interaction energies (kcal mol^−1^) for [*M*I*L*]_4_ (*M* = Ag, Cu) model compounds

	*E*_int_^*AB*^	*E*_cl_^*AB*^	*V*_xc_^*AB*^	*V*_xc_^*AB*^/*E*_int_^*AB*^
Ag1—N1	−132.2	−89.4	−42.7	0.323
Cu1—N1	−173.4	−113.0	−60.4	0.348
Ag1—I1	−74.9	−27.8	−47.1	0.629
Ag1—I2	−73.1	−28.4	−44.7	0.611
Ag1—I3	−71.0	−27.0	−44.0	0.620
Cu1—I1	−90.0	−33.0	−57.0	0.633
Cu1—I2	−79.8	−34.6	−45.2	0.566
Cu1—I3	−68.6	−33.2	−35.4	0.516
Ag1⋯Ag2	15.7	23.5	−7.8	
Cu1⋯Cu2	24.9	27.9	−3.0	
I2⋯N1(Ag)†	51.0	53.5	−2.5	
I3⋯N1(Ag)†	47.9	48.8	−0.9	
I2⋯N1(Cu)†	57.3	61.6	−4.3	
I3⋯N1(Cu)†	54.9	56.2	−1.3	

† I2⋯N1, I3⋯N1 = 3.998, 4.178 (*M* = Ag) and 3.687, 3.993 Å (*M* = Cu).

**Table 5:** Selected IQA total (*E*_int_^*AB*^), electrostatic (*V*_cl_^*AB*^) and exchange-correlation (*V*_xc_^*AB*^) interaction energies (kcal mol^−1^) for *M*Cl*L*_3_ (*M* = Ag, Cu) model compounds

	*E*_int_^*AB*^	*E*_cl_^*AB*^	*V*_xc_^*AB*^	*V*_xc_^*AB*^/*E*_int_^AB^
Ag1—N1	−143.1	−103.3	−39.8	0.278
Cu1—N1	−167.1	−119.5	−47.6	0.285
Ag1—Cl1	−131.1	−59.0	−72.1	0.550
Cu1—Cl1	−135.8	−64.6	−71.2	0.524
Cl1⋯N1(Ag)†	67.5	68.0	−0.5	
N1⋯N7(Ag)†	123.7	124.6	−0.9	
Cl1⋯N1(Cu)†	74.4	75.5	−1.1	
N1⋯N7(Cu)†	134.4	136.0	−1.6	

† Cl1⋯N1, N1⋯N7 = 4.244, 3.599 (*M* = Ag) and 3.826, 3.288 Å (*M* = Cu).
